# Heterochromatin Formation Promotes Longevity and Represses Ribosomal RNA Synthesis

**DOI:** 10.1371/journal.pgen.1002473

**Published:** 2012-01-26

**Authors:** Kimberly Larson, Shian-Jang Yan, Amy Tsurumi, Jacqueline Liu, Jun Zhou, Kriti Gaur, Dongdong Guo, Thomas H. Eickbush, Willis X. Li

**Affiliations:** 1 Department of Biomedical Genetics, University of Rochester Medical Center, Rochester, New York, United States of America; 2 Department of Medicine, University of California San Diego, La Jolla, California, United States of America; 3 Department of Biology, University of Rochester, Rochester, New York, United States of America; Stanford University Medical Center, United States of America

## Abstract

Organismal aging is influenced by a multitude of intrinsic and extrinsic factors, and heterochromatin loss has been proposed to be one of the causes of aging. However, the role of heterochromatin in animal aging has been controversial. Here we show that heterochromatin formation prolongs lifespan and controls ribosomal RNA synthesis in *Drosophila*. Animals with decreased heterochromatin levels exhibit a dramatic shortening of lifespan, whereas increasing heterochromatin prolongs lifespan. The changes in lifespan are associated with changes in muscle integrity. Furthermore, we show that heterochromatin levels decrease with normal aging and that heterochromatin formation is essential for silencing rRNA transcription. Loss of epigenetic silencing and loss of stability of the rDNA locus have previously been implicated in aging of yeast. Taken together, these results suggest that epigenetic preservation of genome stability, especially at the rDNA locus, and repression of unnecessary rRNA synthesis, might be an evolutionarily conserved mechanism for prolonging lifespan.

## Author Summary

Aging is characterized by a progressive decline in vitality and tissue function, leading to the demise of the organism. Many models have been proposed to explain the aging phenomenon. Among the many competing and/or overlapping models is the heterochromatin loss model of aging, which posits that heterochromatin domains (which are set up early in embryogenesis) are gradually lost with aging, resulting in de-repression of silenced genes and aberrant gene expression patterns associated with old age. In this paper, we genetically tested the role of heterochromatin in *Drosophila* aging. We find that heterochromatin levels indeed affect animal lifespan and that heterochromatin represses, among other things, rRNA transcription. Loss of heterochromatin thus leads to an increase in rRNA transcription, a rate-limiting step in ribosome biogenesis and protein synthesis. We suggest that the biological functions of heterochromatin formation include controlling rRNA transcription, which might play an important role in general protein synthesis and animal longevity.

## Introduction

Organismal aging is accompanied by the accumulation of damage to DNA and other macromolecules, and a progressive decline in vitality and tissue function. The underlying mechanisms remain unclear, and many models have been proposed to explain the aging phenomenon. Prominent among these models is the “free radical theory of aging”, which posits that the gradual and collective damage done to biological macromolecules (including DNA and proteins) by reactive oxygen species (ROS) from intrinsic (e.g., metabolism) or extrinsic sources (e.g., radiation), is the major cause of organismal aging [1], [2]. Other competing (although some are overlapping) models of aging include genetically programmed senescence [3], [4], heterochromatin loss [5], telomere shortening [6], genomic instability [7], nutritional intake and growth signaling [8]–[10], to name a few. In the heterochromatin loss model of aging, Villeponteau (1997) has proposed that heterochromatin domains, which are set up early in embryogenesis, are gradually lost with aging, resulting in derepression of silenced genes and aberrant gene expression patterns associated with old age [5].

Experimental tests of the role of heterochromatin formation in animal aging, however, have produced controversial results [11]. On the one hand, cellular senescence is associated with an increase in localized heterochromatin formation in the form of Senescence-Associated Heterochromatin Foci (SAHFs), which are a hallmark of replicative senescence of aged cells in culture, and have also been found in the skin cells of aged animals [12]–[14]. On the other hand, it has been shown that premature aging diseases in human and animal models correlate with global heterochromatin loss [15]–[17].

Heterochromatin is important for chromosomal packaging and segregation, and is thus important for genome stability [18], [19]. Indeed, it has been shown in *Drosophila* that heterochromatin is essential for maintaining the stability of repeated DNA sequences and of the rDNA locus in particular [20]. Loss of heterochromatin causes disruption of nucleolar morphology and formation of extrachromosomal circular (ECC) DNA, which results from an increase in illegitimate recombination at the rDNA locus [20]. Interestingly, disruption of heterochromatin and nucleolar structure, and the consequent increase in ECC DNA, have previously been shown to cause accelerated aging in yeast [21], [22]. These reports suggest a positive role for heterochromatin formation in promoting longevity.

To understand the role of heterochromatin in animal aging, and the underlying molecular mechanisms, we altered heterochromatin levels in *Drosophila* by genetically manipulating Heterochromatin Protein 1 (HP1) levels and JAK/STAT signaling, and assessed the effects on aging. Our results suggest that heterochromatin formation positively contributes to preventing premature aging and suppresses illegitimate recombination of the rDNA locus and unnecessary rRNA synthesis.

## Results/Discussion

### Heterochromatin levels are important for longevity

**Figure 1:**
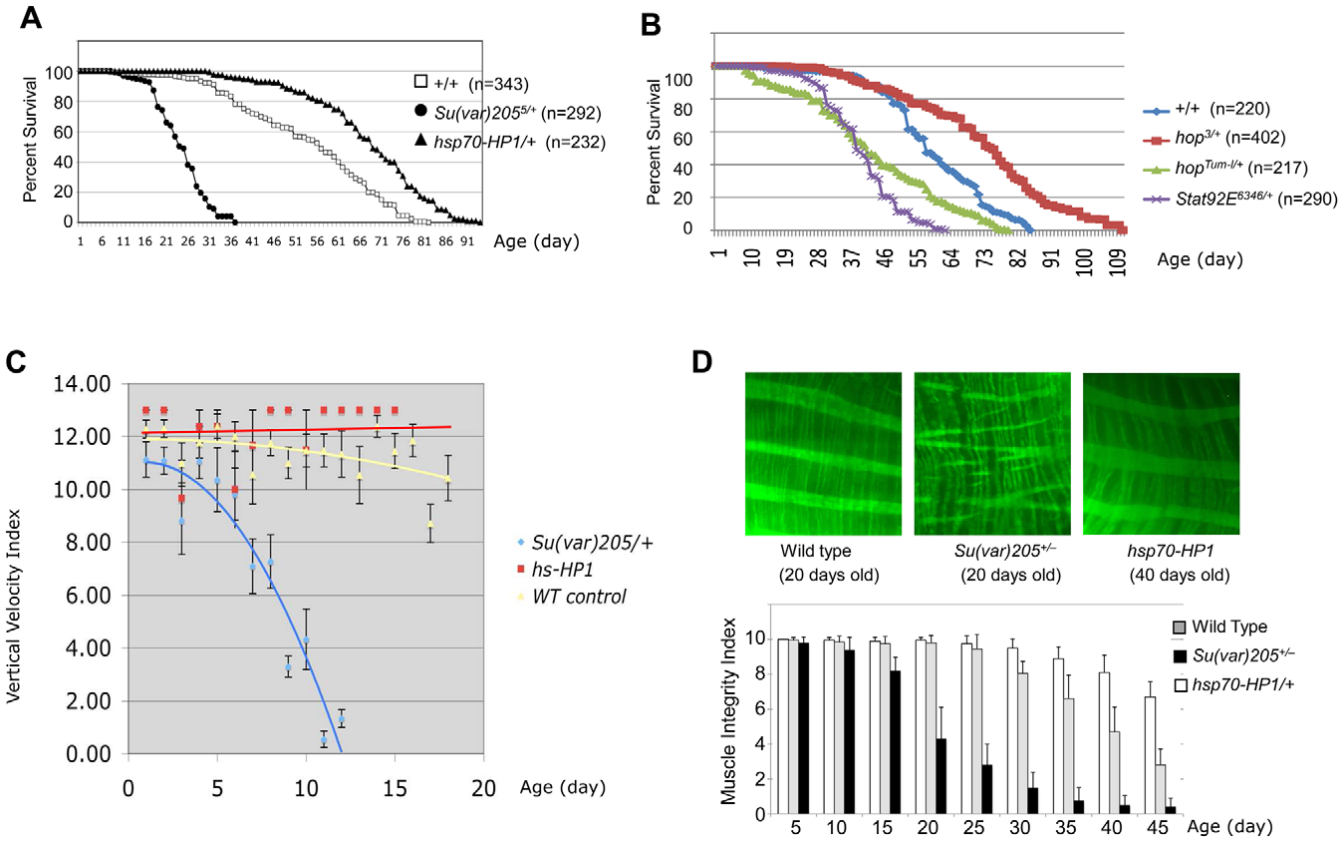
Heterochromatin levels are important for longevity and muscle integrity. Percent survival of adult female flies of indicated genotypes at 25°C. Flies had been made coisogenic by extensive outcrossing (see Methods). n donates the number of flies counted. p values are from Log rank analysis. (A) Flies carrying one copy of *hsp70-HP1* (expressing more HP1) were longer lived (p = 6.31×10^−24^), and flies heterozygous for *Su(var)205^5^* (loss-of-function allele) were shorter lived (p = 2.03×10^−86^) when compared with wild-type “+/+” controls. (B) Flies heterozygous for *hop^Tum-l^* (gain-of-function mutation) and *stat92E^06346^* (loss-of-function allele) were both shorter lived (p = 8.87×10^−23^; 2.92×10^−53^, respectively) than control, and flies heterozygous for *hop^3^* (loss-of-function allele) had longer lifespan compared to control flies (p = 7.34×10^−25^). (C) Flies of indicated genotype and age were confined in food vials and their movements were recorded by video and then analyzed as average velocity. Each data point is the average of >3 recordings of different groups of flies. Error bars are S.E.M. Note that *Su(var)205^+/−^* flies lose mobility precipitously as they age, whereas *hsp70-HP1* flies maintain high mobility for a longer period of time. (D) Top: adult large instestines of indicated age and genotype were stained with phalloidin-fluorescein to reveal the longitudinal and circular intestinal body wall muscle fibers. Images are partions of representative midgut showing 3 longitudinal fibers (wide bands) and circular fibers (thin bands). Note that *Su(var)205^+/−^* flies exhibit premature muscle degeneration: the longitudinal fibers are discontinuous with “loose ends”. Bottom: each fluorescein-stained gut was assigned a morphology score of 0 to 10 based on the integrity of the longitudinal muscle fibers (see Methods). The muscle integrity index was calculated by averaging the scores of 10 guts for each indicated genotype and age. *Su(var)205^5/+^* and *Su(var)205^2/+^* showed similar phenotypes; the results were combined. Error bars are standard deviations.

To investigate whether heterochromatin levels are important for longevity, we examined the life spans of flies with reduced or increased levels of HP1. These flies exhibit reduced or increased levels of heterochromatin, respectively, during development [23], as HP1 is an integral component of heterochromatin and controls heterochromatin levels [24], [25]. We found that reducing HP1 levels by half, as in *Su(var)205^5^* heterozygotes, caused a dramatic shortening of life span compared to isogenic controls (p = 2.03^−86^) (Figure 1A). Similar results were found with a second allele, *Su(var)205^2^* (Figure S1A). Conversely, a moderate overexpression of HP1, caused by basal activity of the *hsp70* promoter, significantly extended life span, resulting in a 23% increase in median life span and a 12% increase in maximum life span (p = 6.31^−24^) (Figure 1A). Similarly, at non-heat shock conditions (25°C), a second (independent) line of *hsp70-HP1* flies also lived significantly longer than their control flies (Figure S1B). At basal levels of transcription, *hsp70-HP1/+* flies exhibited higher heterochromatin levels [26]. By quantitative real-time polymerase chain reaction (qPCR) measurements, we found that these flies had approximately 20% higher HP1 mRNA expression than control (Figure S2A). Over-expression of HP1 at higher levels, such as under heat-shock inducible conditions, however, caused developmental abnormality or lethality to the animal. These results suggest that heterochromatin levels significantly influence life span, and moderately higher levels of heterochromatin promote longevity.

Since both JAK overactivation and STAT loss reduce heterochromatin levels [26], [27], we investigated the effect of altering JAK/STAT signaling on aging. JAK/STAT signaling plays two roles: in the canonical pathway, JAK/STAT directly regulates target gene expression [28], [29], while in its non-canonical function, unphosphorylated STAT is essential for heterochromatin formation [26], [27] and genome stability [19]. In the canonical pathway, loss of STAT has effects equivalent to loss of JAK and opposite to JAK overactivation. However, in the non-canonical function, loss of STAT has the same effects as JAK overactivation, causing heterochromatin destabilization [26], [30] and genome instability [19]. We examined the life span of *Stat92E^+/−^* flies and those heterozygous for gain- or loss-of-function mutations of *hop*. We found that flies heterozygous for either the gain-of-function *hop^Tum-l^* or the *Stat92E* mutation exhibited shortened lifespans compared with wild-type control flies (p = 8.87^−23^; 2.92^−53^, respectively), while flies heterozygous for a loss-of-function *hop* allele, *hop^3^*, had longer lifespans (p = 7.34^−25^) (Figure 1B). These results are consistent with the idea that heterochromatin levels influence lifespan.

A previous study has shown that *Drosophila* life span was only slightly reduced in *Su(var)205* heterozygotes and was not affected by a chromosomal duplication that encompasses the *Su(var)205* locus and many other genes [31]. Using chromosomal duplication, any effects associated with higher levels of *Su(var)205* might be masked by higher levels of other neighboring genes. In studies with the loss-of-function heterozygotes, the authors in that study ensured isogenicity of their compared strains by extensively back-crossing the mutations into a common genetic background, and relied on the suppression of position-effect variegation (PEV) to determine the presence of the *Su(var)205* mutation. PEV results from heterochromatin-mediated gene repression, commonly seen in loss of eye pigmentation [24]. The presence or absence of the *Su(var)205* mutation was assumed to correlate 100% with the PEV phenotype. However, we have found that this is not the case. We examined the PEV phenotype of *w^m4^* in the progeny of single pairs of *w^m4^; Su(var)205^5^/CyO* and *w* flies, and found that the PEV phenotype was not 100% correlated with the *Su(var)205* mutation (Figure S3), rather, there was less PEV than would be expected, suggesting that, with regard to suppression of PEV, either the *Su(var)205* mutation is not completely penetrant or that there is an incomplete epigenetic reprogramming at the *w^m4^* locus, or both. On the other hand, it has been shown that many *Su(var)* mutations exhibit maternal-effect suppression of PEV [32], such that the PEV phenotype can be modified regardless of inheritance of the *Su(var)* mutation. It has also been shown that HP1 mutations disrupt epigenetic reprogramming, causing transgenerational inheritance of epigenetic information [33], [34]. Thus, in the aging study by Frankel and Rogina (2005) [31], the presence or absence of the *Su(var)205^2^* mutation in the test flies may not have been accurately determined. In our current studies, we confirmed the presence of *Su(var)205* mutations in the coisogenic strains by both suppression of PEV and homozygous lethality (see Materials and Methods). We found that heterochromatin levels are essential for longevity using both gain- and loss-of-function strategies.

### Heterochromatin levels are essential for the maintenance of adult muscle integrity and function

To investigate the cause of altered life span in flies with different heterochromatin levels, we observed the behaviors of these flies by video-recording (see Methods). Video playbacks show that aged flies exhibited a gradual loss of mobility and eventually became immobile (Videos S1, S2, S3). By quantifying their mobility (see Methods), we found that, compared with wild-type controls, flies with reduced heterochromatin levels lost mobility much faster, and those with increased heterochromatin levels maintained their mobility for a longer period of time (Figure 1C; Videos S1, S2, S3).

It has been shown in *C. elegans* and *Drosophila* that old animals die of sarcopenia (muscle degeneration) [35] and impaired muscle function precedes aging [36], similar to the gradual loss of muscle function and frailty in aging humans. Since we found that heterochromatin levels influence *Drosophila* life span, and since the altered life span was associated with the animals' mobility, we investigated whether loss of heterochromatin is associated with muscle degeneration.

We used whole-mount fluorescent immunostaining to examine the integrity of the large intestinal wall muscle, which can be visualized readily in adult flies of different ages after minimal dissection. The fly large intestinal wall muscles consist of longitudinal (thick) and circular (thin) muscle fibers (Figure 1D, top left). We found that, wild-type flies exhibited progressive muscle degeneration as they aged (sarcopenia), such that the gut muscle fibers gradually showed breakage starting around day 20, and extensive breakage was seen in 40-day-old fly gut muscles. We found that heterochromatin levels affected the ability to maintain muscle integrity, with 20-day-old *Su(var)205^+/−^* flies showing extensive muscle fiber breakage (Figure 1D, top middle), whereas *hsp70-HP1* flies maintained their muscle integrity beyond 40 days after eclosion (Figure 1D, top right). We quantified the breakages in longitudinal muscle fibers in a defined area of the midgut and calculated the muscle integrity index for each genotype and age (see Methods). We found that the muscle integrity indices correlate well with the mobility of flies of different genotype and age (Figure 1D, bottom). These results are consistent with the differences in fly motility that we directly observed. Thus, maintenance of heterochromatin levels is essential for the maintenance of muscle structure and function, which consequently affect animal mobility and lifespan.

### Heterochromatin levels decline with aging

**Figure 2:**
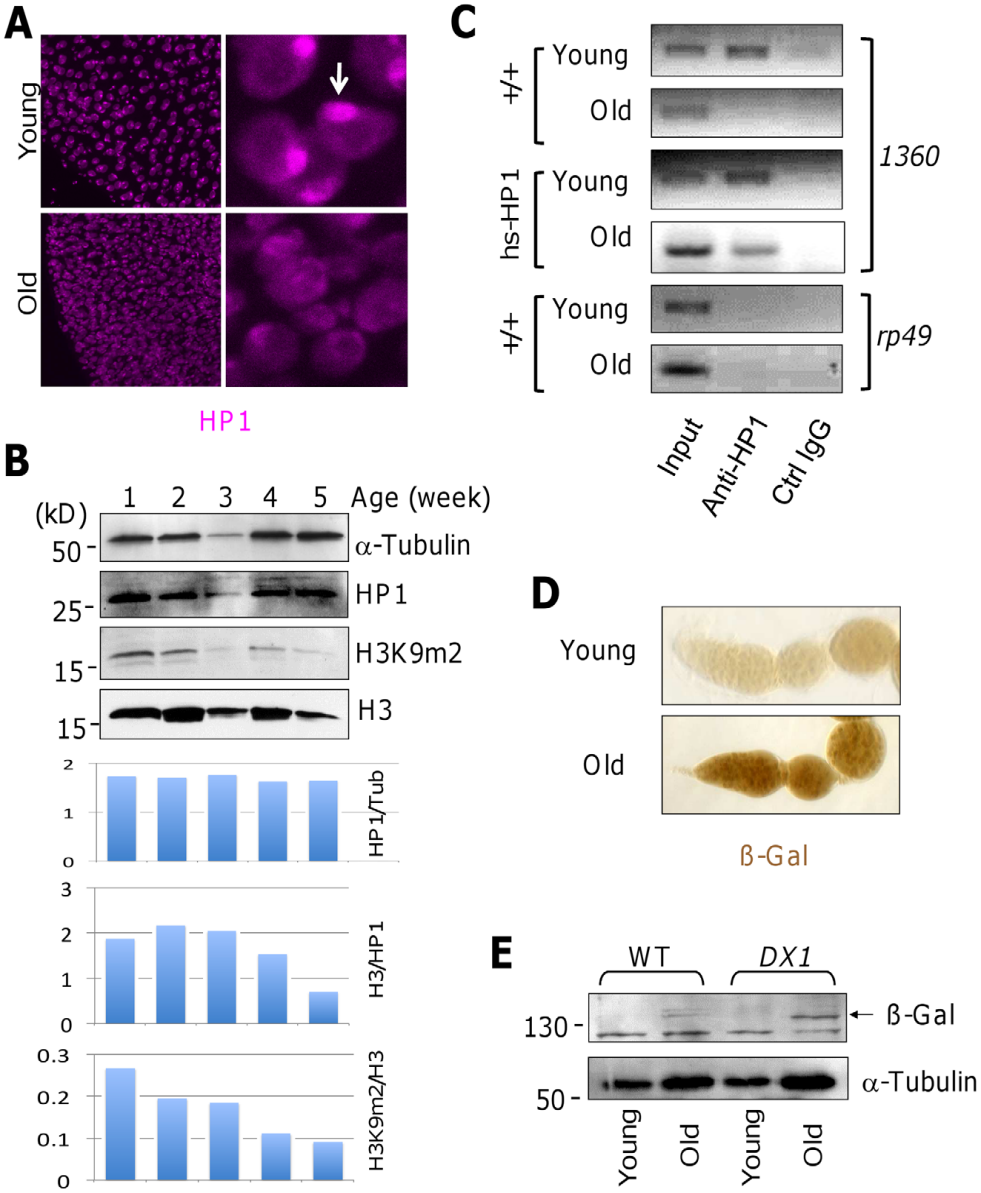
Heterochromatin levels decline with aging. (A) Gut tissues from young (3-day old) and old (35-day old) female flies were dissected and stained with anti-HP1 (magenta). Images were scanned at identical settings with confocal microscopy. Note that HP1 forms prominent foci in the young gut (one pointed by an arrow), whereas in the old gut HP1 staining seems more diffuse and lacks the prominent foci. (B) Male flies of indicated age (in weeks) were homogenized and the protein extracts were subjected to SDS-PAGE and blotted sequentially with antibodies for H3K9m2, HP1, H3, and α-Tubulin. Representative images for one of the three experiments are shown. Lower panels show intensity ratios as indicated. Note that total H3 levels decrease with age, and that H3K9m2 signals decrease with age even when normalized to total H3. (C) Chromatin immunoprecipitation (ChIP) was carried out with anti-HP1 antibodies using extracts from young (3-day old) and old (35-day old) male wild-type or *hsp70-HP1/+* flies. Note that HP1 is enriched in *1360* (representative heterochromatin sequence) of young but not old wild-type flies (lane 2, top two panels), and is detectable in both young and old *hsp70-HP1/+* flies (middle panels). (D) Ovaries from young (3-day old) and old (35-day old) female *DX1* flies were stained with anti-ßgal. Note the much increased ßgal levels in old ovaries. (E) Total protein from single young (3-day old) and old (35-day old) female flies of indicated genotypes were blotted by anti-ß-gal antibodies or anti-aTublin (control). Note the appearance of ß-gal in old *DX1* flies (arrow). The band below ß-gal is a nonspecific band.

If heterochromatin levels are important for longevity and tissue integrity, then normal aging should be accompanied by gradually decreasing heterochromatin levels. Indeed, it has been shown that normal aging in *C. elegans*, as well as the premature aging observed in human progeric syndromes, is correlated with changes in nuclear architecture and loss of heterochromatin [15]–[17], [37]. Since pericentromeric heterochromatin is readily observable in enterocytes, we examined HP1 foci in enterocytes of young and old adult flies. We found that, in contrast to young flies, whose enterocytes had prominent chromocenter enriched with HP1 (Figure 2A; top), old flies had much reduced levels of heterochromatin, with many nuclei in the gut epithelia lacking pronounced HP1 foci (Figure 2A; bottom). Since HP1 is recruited to heterochromatin by binding to histone H3 di- or tri-methylated at lys9 (H3K9m2 or H3K9m3), heterochromatin-specific chromatin modifications, we further investigated changes in the levels of H3K9m2 in flies of different ages. Interestingly, we found that total histone H3 levels decreased with age when compared with the non-histone nuclear protein HP1, which remained nearly constant relative to α-tubulin (Figure 2B). However, total levels of H3K9m2 showed a more dramatic decrease with age, and the decrease was obvious even relative to H3 levels (Figure 2B). Total H3K4m3 levels, on the other hand, showed a less dramatic decrease (Figure S4). Our results are consistent with previous reports that the levels of total histone H3 and heterochromatin marks decrease when animals age [15]–[17], [38], [39].

Taken together, the above observations suggest that, total histone H3 levels and their modifications by methylation, especially methylation of K9, exhibit gradual decline when animals age. The presence of excess HP1 throughout life might help preserving H3K9 methylation, thus delaying its decline. Although HP1 protein levels control heterochromatin levels during development, HP1 is not the sole factor determining heterochromatin formation post-development, especially in aged adult flies, where we have observed a decrease in the levels of H3K9m2, but not of HP1.

To further confirm that HP1 is not localized on heterochromatin sequences in old flies, we carried out chromatin immunoprecipitation (ChIP) experiments to determine HP1 occupancy on the transposable element *1360*, which is highly enriched in constitutive heterochromatin [40]. Transposable element *1360* is present in >300 copies in the fly genome and has been used as a representative sequence for global heterochromatin [26], [40]. The *ribosomal protein 49* (*rp49*) gene is a constitutively transcribed gene normally not associated with heterochromatin and can be used as a negative control. By performing ChIP experiments using anti-HP1 antibodies followed by PCR amplification, we assessed the levels of HP1 occupancy in these sequences. Indeed, we found that HP1 was found associated with *1360* in young but not old wild-type flies (Figure 2C), whereas in flies carrying the *hsp70-HP1* transgene, HP1 was also found associated with *1360* even in old flies. These results are consistent with the idea that heterochromatin levels decrease with aging, and that over-expressing HP1 prevented heterochromatin decline. Taken together, these results suggest that there is a gradual loss of heterochromatin when flies age, as with *C. elegans* and humans, that the lack of HP1 localization to heterochromatin foci in old flies is likely due to the loss of H3K9 methylation, and that over-expression of HP1 throughout life can prevent or delay heterochromatin loss.

Heterochromatin loss could cause re-expression of genes that are normally repressed by heterochromatin. We thus examined the expression of a heterochromatinized *lacZ* transgene in young and old *DX1* flies. These flies carry a tandem array of seven *P[lac-w]* transgenes, but *lacZ* (and *white^+^*) expression from these transgenes is normally repressed by DNA repeat-induced heterochromatin formation [41]. A reduction in heterochromatin can cause derepression of the *lacZ* gene contained in the *P[lac-w]* elements of *DX1* flies [27], [41]. Indeed, we found that old, but not young, *DX1* flies expressed *lacZ* (Figure 2D, 2E), consistent with the idea that heterochromatin levels decline with age. Thus, normal aging is accompanied by a gradual loss of heterochromatin in *Drosophila* as well.

### Heterochromatin is essential for maintaining nucleolar stability

**Figure 3:**
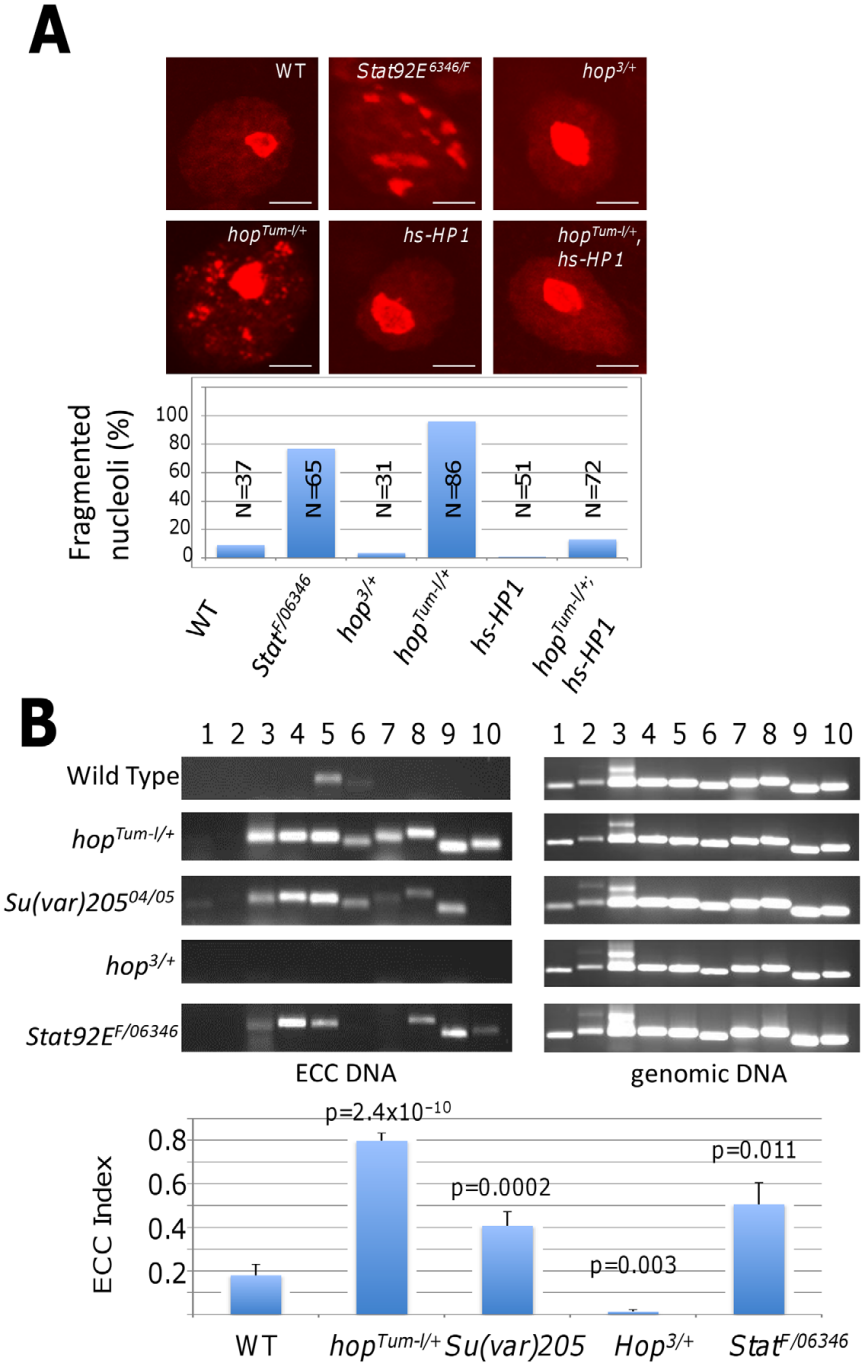
Heterochromatin affects stability of the nucleolus and rDNA locus. (A) 3^rd^ instar larval salivary glands of indicated genotypes were stained with anti-Fibrillarin. *Stat92E^F^* is a hypomorphic allele. Images were scanned with a confocal microscope at identical settings, and representative nuclei and quantifications are shown. N represents the number of nuclei scored. (B) 3^rd^ instar larvae of indicated genotypes were processed for ECC (left) or genomic (right; control) DNA. The presence of indicated sequences were detected by PCR. *Rp49* (lane 1) and *5S rDNA* (lane 2) are non-repeated sequences and are used as negative controls for ECC. 1: rp49. 2: 5S rDNA. 3: Satellite 1.688. 4: 5′ 18S rDNA. 5: 3′ 18S rDNA. 6: 18 to 5.8S spacer. 7: 5.8S rDNA. 8: 28S rDNA. 9: Mid 28S rDNA. 10: 3′ 28S rDNA. Each genotype was analyzed three times; representative PCR results are shown. ECC levels were quantified by calculating the ECC index for each genotype (see Methods) and the results are shown (bottom). Error bars indicate S.E.M., and p values (Student's *t*-Test) indicate statistical significance of the differences compared with wild type. Note that ECC DNA (lane 3–10) was detected at high levels in *Stat92E*, *Su(var)205*, and *hop^Tum-l^* heterozygotes, only minimally in wild-type controls, but not in *hop* loss-of-function heterozygotes.

We next investigated the possible mechanism(s) by which heterochromatin formation promotes life span extension. It has been shown previously that H3K9 methylation and RNA interference regulate nucleolar stability [20]. Loss of HP1 or Su(var)3–9 levels causes fragmentation of the nucleolus, as revealed by the nucleolar marker Fibrillarin [20]. When we examined the effects of heterochromatin levels on nucleolar morphology, which can be seen most easily in 3^rd^ instar larval salivary gland giant nuclei, we found that conditions that decreased heterochromatin levels, such as JAK over-activation [27] or loss of STAT [26], were associated with nucleolar instability (Figure 3A). Conversely, conditions that increased heterochromatin formation, such as *hop* loss-of-function or HP1 over-expression [27], were associated with a stable nucleolus: the presence of a single, round nucleolus (Figure 3A). Moreover, HP1 over-expression suppressed the nucleolar fragmentation associated with *hop^Tum-l^* (Figure 3A). These results are consistent with previous findings that JAK overactivation disrupts heterochromatin formation and that heterochromatin formation is important for nucleolar stability [20], [27].

Nucleolar fragmentation has been attributed to illegitimate recombination of repeated DNA sequences, resulting in instability of the highly repeated rDNA locus. Illegitimate recombination events can be assessed quantitatively by measuring the levels of extrachromosomal circular (ECC) DNA [20]. We isolated ECC DNA from flies of different genotypes and quantified ECC levels by calculating ECC index (see Methods). Indeed, we found increased ECC levels in mutants with decreased heterochromatin, such as *hop^Tum-l^*, *Stat92E*, and *Su(var)205*, and decreased ECC levels in mutants with increased heterochromatin formation, such as *hop^+/−^* (Figure 3B).

Interestingly, increased ECC formation due to instability of the rDNA locus has previously been shown to cause accelerated aging in yeast [21], [22]. Taken together with our results from *Drosophila*, we suggest that the rDNA locus (or nucleolus) might be an important cellular target regulated by heterochromatin formation.

### Heterochromatin controls rRNA synthesis

**Figure 4:**
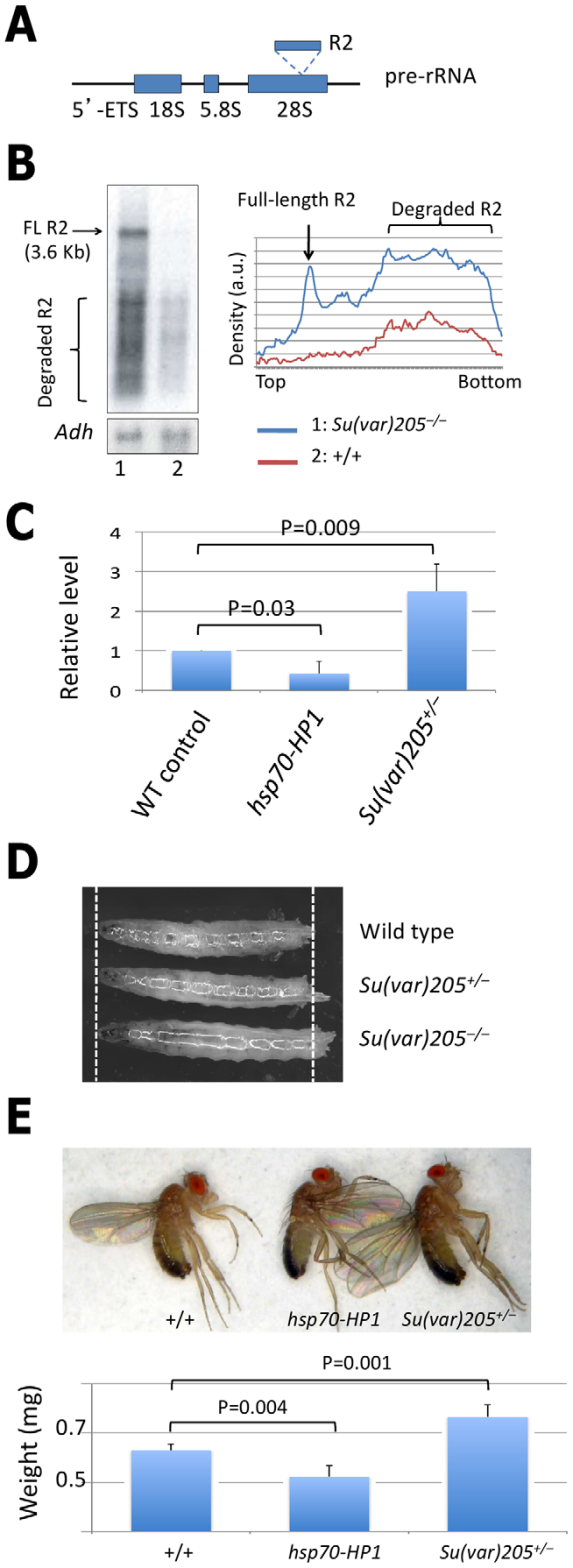
Heterochromatin controls rRNA transcription. (A) Schematic representation of a pre-rRNA transcript, with an R2 element inserted into the 28S gene. Arrows above the 5′ETS region represent PCR primers used for qPCR analysis. (B) RNA was isolated from 3^rd^ instar larvae of indicated genotypes and was subjected to Northern blotting with an R2 5′ antisense probe. Transcripts from the *Adh* gene were used as a loading control. Levels of the transcripts were quantified with a phospho-imager. The full-length (FL) R2 transcript is 3.6 kb. The lower bands are all degradation products, which appear soon after transcription [42]. A density plot is shown to the right with arbitrary units (a.u.). (C) The levels of pre-rRNA in 2-day-old male flies of indicated genotypes were measured by qRT-PCR. Relative pre-rRNA levels are shown with standard deviations. (D) Representative larvae of indicated genotypes. *Su(var)205^−/−^* were transheterozygous for *Su(var)205^2^* and *Su(var)205^5^*. (E) Flies of indicated genotypes were outcrossed to minimize genetic background effects and were raised in parallel at 25°C with similar larval density. Top: Representative male flies of indicated genotypes. Bottom: The fly body weight was measured as the average of 10 2-day old male flies. *Su(var)205^2/+^* and *Su(var)205^5/+^* male flies had similar body weights, the results were combined and shown as *Su(var)205^+/−^*. Standard deviations and p values (compared with wild-type control; Student's *t*-Test) are shown.

Finally, we investigated the functional consequences of nucleolar instability. The nucleolus is the site of rRNA biogenesis, where precursor rRNA molecules are transcribed and processed to give rise to 18S, 5.8S and 28S rRNAs (Figure 4A). In *Drosophila*, as well as in mammals, the rDNA locus consists of a few hundred rRNA transcriptional units in tandem repeats. The number of rDNA genes vastly exceeds what is needed for adequate rRNA transcription. Normally only 20 to 25 units (<10% of the total) are actively transcribed, while the majority of the rDNA locus is silenced presumably by unknown epigenetic mechanisms [42]. Since it has been shown that loss of heterochromatin, as in *Su(var)205* transheterozygotes, leads to illegitimate recombination and thus instability of the rDNA locus [20], we investigated whether heterochromatin loss also leads to derepression of rDNA transcription.

The levels of rDNA transcription can be more sensitively detected by examining the transcription of a class of transposons (e.g., R2 elements) that are specifically inserted into, and are cotranscribed with, the 28S rDNA gene [42]. Normally the host “selects” a region of the rDNA locus free of R2 insertions for transcription and represses the R2-inserted rDNA units by unknown mechanisms [42]. We found that in *Su(var)205* transheterozygous mutants, however, transcription of R2 elements was dramatically increased by >40 fold compared to their sibling heterozygous control flies (Figure 4B). This suggests that in preserving the structural integrity of the nucleolus, heterochromatin formation plays a crucial role in silencing the transcription of the majority of rDNA genes, and that loss of heterochromatin causes a dramatic increase in rRNA transcription, which could lead to an increased capacity for protein synthesis, conducive to growth and accelerated aging.

To investigate whether the moderately altered heterochromatin levels that have been shown to alter lifespan (Figure 1), affect the rate of rRNA synthesis, we measured pre-rRNA transcript levels in flies heterozygous for *Su(var)205* or carrying an *hsp70-HP1* transgene by quantitative real-time PCR (qRT-PCR). Indeed, we found that flies in which HP1 is moderately over-expressed (by basal activity of the *hsp70* promoter without heat shock) contained >50% less pre-rRNA, whereas *Su(var)205* heterozygous flies had levels of pre-rRNA transcripts that were >2 fold higher (Figure 4C).

To determine whether these altered rRNA transcription rates affect growth, and thus the body size of the adult fly, which has often been inversely associated with lifespan [43], we measured the body size and weight of larval and adult flies with altered heterochromatin levels as mentioned above. Indeed, we found that flies moderately over-expressing HP1 had a smaller body weight, and that *Su(var)205^−/−^* larvae had a larger body size (length) (Figure 4D), and that *Su(var)205^+/−^* flies had a larger body weight (Figure 4E). The differences in body weight were not as pronounced as those in rRNA transcription, suggesting that body size may not be solely regulated by the rRNA transcription rate. Nonetheless, these results are consistent with the idea that changes in the rate of rRNA transcription may impact global protein synthesis and thus the growth of the organism.

### Concluding remarks

In summary, we have found that heterochromatin formation promotes longevity, genome stability, and suppresses rRNA transcription. The causal relationship between aging and rRNA transcription, however, awaits further investigation. It is interesting to note that factors that promote growth, such as insulin signaling and protein synthesis, usually accelerate aging, whereas inhibition of these pathways extends life span [9], [44]–[49]. Moreover, it has been shown in yeast that the Sir2 histone deacetylase counteracts aging by inducing heterochromatin formation at the rDNA locus [21], [22], thereby suppressing rRNA transcription and maintaining stability of the rDNA locus. In mammals, it has been shown that heterochromatin and Sirt1 epigenetically silence rDNA transcription in response to intracellular energy status [50]. Thus, loss of rDNA silencing due to heterochromatin loss could lead to instability and increased rRNA transcription, which promotes protein synthesis in general. We suggest that suppression of rDNA transcription might be an evolutionarily conserved mechanism essential for animal longevity.

## Materials and Methods

### Fly stocks and genetics

All crosses were carried out at 25°C on standard cornmeal/agar medium unless otherwise specified. Fly stocks of *hop^Tum-l^*, *Stat92E^06346^*, *Su(var)205^05^*, *Su(var)205^02^*, *hop^3^*, *hsp70-Gal4*, and *UAS-eGFP* were from the Bloomington *Drosophila* Stock Center (Bloomington, IN). Fly stocks of *DX1* and *6-2* mini-*white^+^* (J. Birchler), and *hsp70-HP1* (G. Reuter; L. Wallrath) were generous gifts. All alleles used for life span analyses were extensively outcrossed before experiments (see below).

### Life span analysis

The following outcrossing schemes were used to minimize genetic background effects. *hsp70-HP1* (line 1; *p[hsp70-HP1-eGFP, ry^+^]* carried on the 2^nd^ chromosome; [51]) flies were outcrossed to a *ry^506^* stock for ten generations, and the *ry^+^* or *CyO* marker was followed to derive outcrossed *hsp70-HP1/+* and *CyO/+* flies, respectively. These flies were crossed to establish new “outcrossed” *hsp70-HP1 (p[ry^+^])/CyO; ry^506^* flies. *Su(var)205^05^/CyO* flies were outcrossed to *In(1)w^m4^* stock for ten generations, and the *CyO* marker or suppression of *In(1)w^m4^* PEV was followed to derive outcrossed *In(1)w^m4^*; *Su(var)205^05^/+* and *In(1)w^m4^*; *CyO/+* flies, respectively. These flies were crossed in single pairs to derive new “outcrossed” *In(1)w^m4^*; *Su(var)205^05^/CyO* stocks (the presence of *Su(var)205^05^* was confirmed by both suppression of PEV and homozygous lethality). For lifespan analysis, the “outcrossed” *hsp70-HP1 (p[ry^+^])/CyO; ry^506^* flies were crossed to *In(1)w^m4^* flies, and the “outcrossed” *In(1)w^m4^*; *Su(var)205^05^/CyO* flies were crossed to *ry^506^* flies, and the F1 non-*CyO* flies were collected for lifespan analysis. “Wild-type” control flies were the F1 of *In(1)w^m4^* and *ry^506^* flies. An independent stock of *hsp70-HP1* (line 2; an unmarked *p[hsp70-HP1-lacI]* inserted in the 2^nd^ chromosome; [52]) was outcrossed to *In(1)w^m4^* stock (with a *CyO* chromosome “floating”) for ten generations. An isogenic line of *In(1)w^m4^*; *hsp70-HP1* (line 2)/CyO flies was established from a single male, and the presence of *hsp70-HP1* was confirmed by its strong enhancement of PEV. This stock was used for lifespan studies, and a line that did not enhance PEV (which was considered not carrying the *hsp70-HP1* transgene) was used as wild-type control.

To assess life span, 2-day-old females were separated from males and were transferred to fresh vials at 20 flies/vial, and were subsequently transferred to fresh vials every 2–3 days. Dead flies were counted upon each transfer. Only female heterozygotes were analyzed because *hop* is located on the X chromosome and the hemizygous mutants are not viable. Flies were cultured at 25°C and 70% humidity.

### Immunostaining, ChIP, and Western blots

Mouse monoclonal anti-HP1 (C1A9; Developmental Hybridoma Bank, Iowa; 1∶200), rabbit anti-H3(di)mK9 (07-212; Upstate Biotechnology; 1∶200), rabbit anti-GFP (CloneTech; ), and rabbit anti-Fibrillarin (Abcam; 1∶500) were used as primary antibodies and fluorescent secondary antibodies (Molecular Probes) were used in whole-mount immunostaining. Tissues were fixed in 4% paraformaldehyde/PBS and 0.3% Triton-X/PBS. Stained tissues were photographed with a Leica confocal microscope. Images were cropped and minimally processed using Adobe Photoshop CS.

For chromatin immunoprecipitation (ChIP), adult flies of appropriate ages were snap frozen with liquid nitrogen, and then were cross-linked with 1.8% formaldehyde. The flies were homogenized in cell lysis buffer, and ChIP was performed as previously described [26].

### Extrachromosomal Circular (ECC) DNA measurement

Adult flies of appropriate genotypes were split into two groups, one for Hirt ECC DNA isolation as described [20], and the other for genomic DNA isolation by standard protocols to use as controls. Typically 10 adult males were ground in 500 µl Hirt lysus buffer (0.6% SDS, 10 mM EDTA, pH 8.0) for ECC isolation or in 200 µl Buffer A (0.5% SDS, 100 mM EDTA, 100 mM NaCl, 100 mM Tris-HCl, pH 7.5) for genomic DNA isolation. Genomic DNA was quantified by spectrometry, and 100 ng of genomic DNA and an equal volume of ECC DNA were used for PCR amplification. Primer sequences used for amplifying ECC and control DNA were as previously reported [20]. The PCR products from ECC sample and genomic control sample were loaded on the same agarose gel. PCR bands were revealed by ethidium bromide staining and photographed. The level of ECC was measured as the ratio of ECC band intensity to that from genomic control run on the same gel. Three independent experiments were done for each genotype. The ECC index was calculated as the sum of each ECC/genomic ratio divided by the total number of ECC bands:
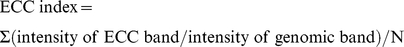
Where N denotes the total number of ECC species examined (N = 8 in this experiment).

### Analyses of adult fly motility and intestinal muscle integrity

Flies were outcrossed as described in “Life span analysis” to minimize genetic background effects. Virgin female flies of a particular genotype were grouped by 5 in a cornmeal food vial (without supplementing yeast) and were passed daily to a fresh vial. Flies of different ages were video recorded for 2 min, around 9:30 AM, with a mounted digital camera. Recording was started after flies fell to the bottom of the vial by knocking the vial on a bench top. Motility scores were assigned to each fly in the video playback according to the speed with which it moved upward in the vial.

Adult flies of desired ages were dissected and fixed with formaldehyde. The intestines were stained with phalloidin-fluorescein and observed with an epifluorescence microscope. At least 10 flies of each genotype and age were dissected. Each fluorescein-stained large intestine was assigned a Morphology Score (MS) of 0 to 10 based on the number of breakages in the longitudinal muscle fibers in a 3 gut-diameter long stretch of the midgut immediately adjacent to the hindgut. MS = 10−n, where n = the number of breakages in the defined region of the midgut. A score 10 represents prefect morphology: no muscle fiber breakage in the longitudinal muscles (n = 0). When n≥10, usually no continuous longitudinal muscle fibers can be identified and accurate counting breakages becomes difficult. In this case, a Morphology Score of 0 was assigned. So MS represents the worst morphology. The muscle Integrity Index is defined as the total MS of each genotype and age divided by the total number of intestines observed (N).
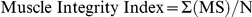



### Pre-rRNA measurement by qRT–PCR

Total RNA was isolated from 10 adult male flies (2-day-old) of desired genotypes using the RNeasy Mini Kit (Qiagen) or trizol (Invitrogen) according to the manufacturer's instructions. One µg of total RNA and primers specific for pre-rRNA and rp49 (control) were used to make the first strand cDNA using Superscript III reverse transcriptase (Invitrogen) in 50 µl total reaction volume. The cDNA (at 1∶100 dilution) was used as template for qRT-PCR analysis using SYBR green based detection on a BioRad iCycler. Reactions were carried out in triplicate, and melting curves were examined to ensure the presence of single products. The levels of pre-rRNA were quantified relative to *rp49* transcript levels (control) and were normalized to a wild-type control. The following primer pairs (forward and reverse) were used.

*rp49*: tcctaccagcttcaagatgac, cacgttgtgcaccaggaact

*pre-rRNA (5′ETS)*: atcggccgtattcgaatggattta, ctactggcaggatcaaccaga

## Supporting Information

Figure S1 Life span of flies with altered HP1 levels. Percent survival of adult female flies of indicated genotypes. Flies had been made coisogenic by extensive outcrossing (see Methods). n donates the number of flies counted. p values are from Log rank analysis. All experiments were carried out at 25°C (with heat-shock), where transgenes were expressed at basal levels. (A) Percent survival of *Su(var)205^2^* heterozygous flies and their wild-type control flies. (B) Life spans of flies carrying one copy of *hsp70-HP1* (line 2) and their wild-type “+/+” controls.
(TIF)

Figure S2 Quantification of HP1 mRNA levels. Total RNA was isolated from young (2 to 3-day old) and old (35 to 36-day old) wild-type or *hsp70-HP1/+* male flies, and the samples were subjected to quantitative real-time PCR analysis for *HP1* or *rp49* (control) mRNA levels. *HP1* expression was normalized to that of *rp49*, and the results were normalized to wild-type young males. Note that *hsp70-HP1/+* flies express moderately higher levels of HP1 than wild-type.
(TIF)

Figure S3 Correlation between *Su(var)205* mutation and PEV suppression. Examples of F1 progeny flies from single pairs of *w^m4^; Su(var)205^5^/CyO* virgin females and *w^1118^* males. Flies #1, 2, and 4 have identical genotype: *w^m4^/w^1118^; CyO/+*. Fly #3 has the genotype of *w^m4^/w^1118^; Su(var)205^5^/+*. Note fly #1 shows the normal PEV phenotype (decreased eye pigmentation), while flies #2 to 4 all exhibit suppressed PEV (eyes dark red). In the F1 progeny of single pairs of *w^m4^; Su(var)205^5^/CyO* virgin females and *w^1118^* males, 58% (n = 49/84) of *w^m4^/w^1118^; CyO/+* female progeny flies (without inheriting *Su(var)205^5^*) showed suppressed PEV phenotype, with the eye color dark red, indistinguishable from their *w^m4^/w^1118^; Su(var)205^5^/+*siblings.
(TIF)

Figure S4 Levels of H3K4m3 at different ages. Male flies of indicated age (in days) were homogenized and the protein extracts were subjected to SDS-PAGE and blotted sequentially with antibodies for H3K4m3, HP1, and total H3. Representative images for one of the three experiments are shown. Intensity ratios are shown. Note that relative to HP1, total H3 levels decrease with age, and that H3K4m3 signals (relative to H3) also show moderate decrease with age.
(TIF)

Video S1 Wild-type fly motility. Five 3-day old wild-type control female flies were tested for motility. See Methods for more description of procedures.
(MP4)

Video S2 *Su(var)205^5/+^* fly motility. Five 3-day old *Su(var)205^5/+^* female flies were tested for motility. See Methods for more description of procedures.
(MP4)

Video S3 *hsp70-HP1/+* fly motility. Five 3-day old *hsp70-HP1/+* female flies were tested for motility. See Methods for more description of procedures.
(MP4)
