# Supplementary figures and images for: Heterochromatin Formation Promotes Longevity and Represses Ribosomal RNA Synthesis
Source: PLoS Genet. 2012 Jan 26;8(1):e1002473. doi: 10.1371/journal.pgen.1002473 (PMC3266895; doi:10.1371/journal.pgen.1002473)

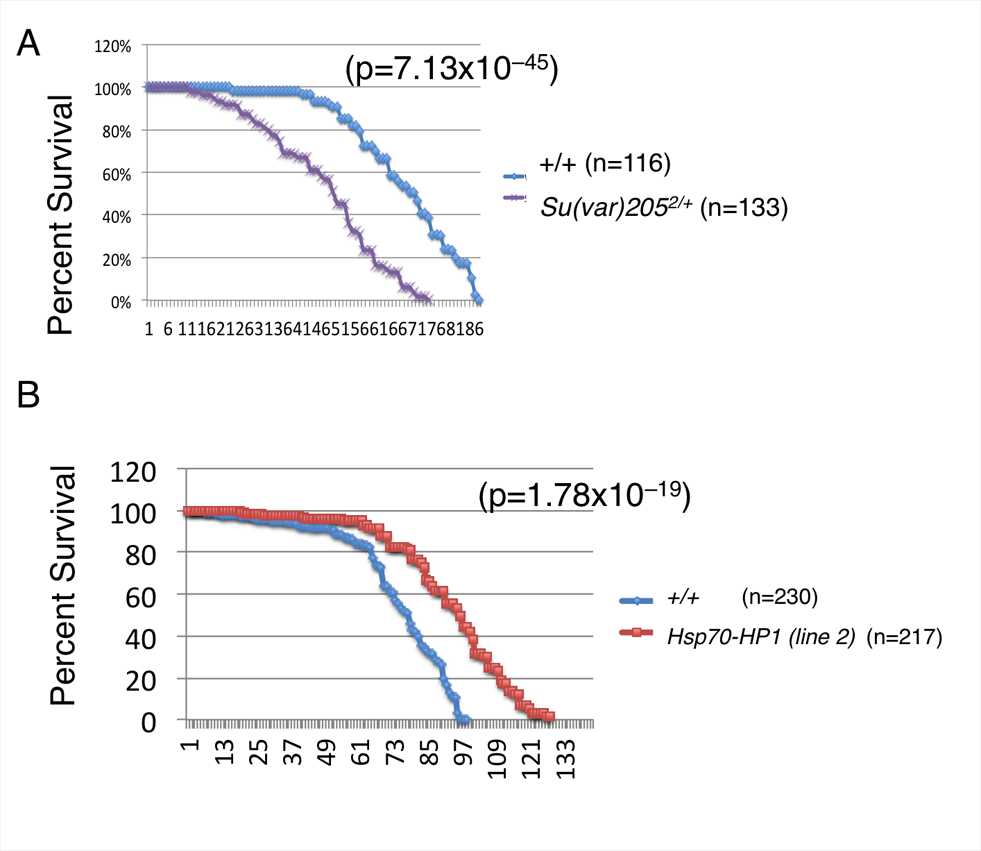

Supplement: Figure S1 — Life span of flies with altered HP1 levels. Percent survival of adult female flies of indicated genotypes. Flies had been made coisogenic by extensive outcrossing (see Methods). n donates the number of flies counted. p values are from Log rank analysis. All experiments were carried out at 25°C (with heat-shock), where transgenes were expressed at basal levels. (A) Percent survival of Su(var)2052 heterozygous flies and their wild-type control flies. (B) Life spans of flies carrying one copy of hsp70-HP1 (line 2) and their wild-type “+/+” controls. (TIF) [file pgen.1002473.s001.tif]

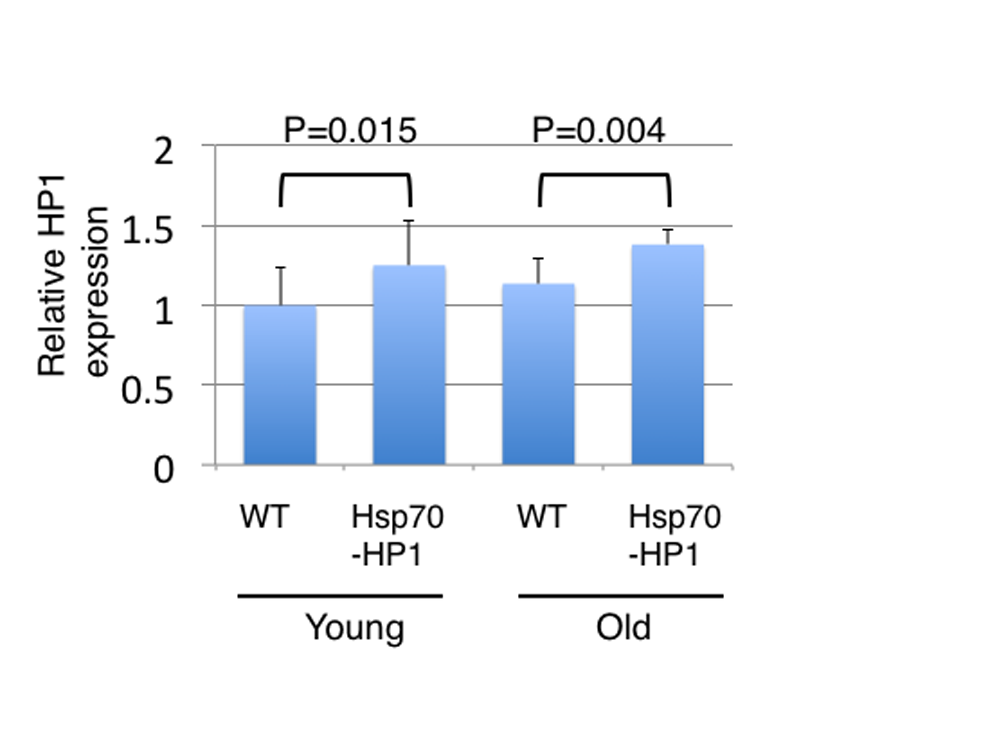

Supplement: Figure S2 — Quantification of HP1 mRNA levels. Total RNA was isolated from young (2 to 3-day old) and old (35 to 36-day old) wild-type or hsp70-HP1/+ male flies, and the samples were subjected to quantitative real-time PCR analysis for HP1 or rp49 (control) mRNA levels. HP1 expression was normalized to that of rp49, and the results were normalized to wild-type young males. Note that hsp70-HP1/+ flies express moderately higher levels of HP1 than wild-type. (TIF) [file pgen.1002473.s002.tif]

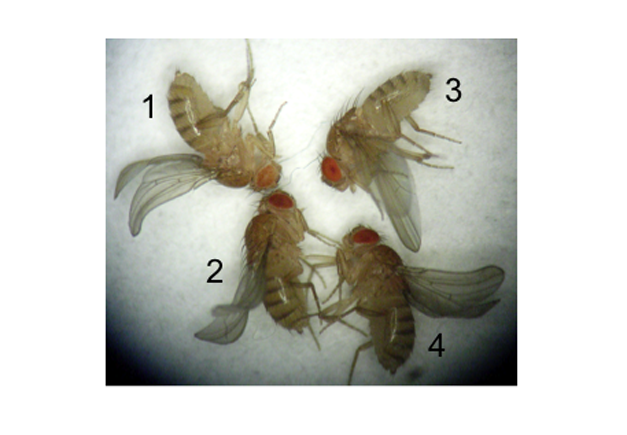

Supplement: Figure S3 — Correlation between Su(var)205 mutation and PEV suppression. Examples of F1 progeny flies from single pairs of wm4; Su(var)2055/CyO virgin females and w1118 males. Flies #1, 2, and 4 have identical genotype: wm4/w1118; CyO/+. Fly #3 has the genotype of wm4/w1118; Su(var)2055/+. Note fly #1 shows the normal PEV phenotype (decreased eye pigmentation), while flies #2 to 4 all exhibit suppressed PEV (eyes dark red). In the F1 progeny of single pairs of wm4; Su(var)2055/CyO virgin females and w1118 males, 58% (n = 49/84) of wm4/w1118; CyO/+ female progeny flies (without inheriting Su(var)2055) showed suppressed PEV phenotype, with the eye color dark red, indistinguishable from their wm4/w1118; Su(var)2055/+siblings. (TIF) [file pgen.1002473.s003.tif]

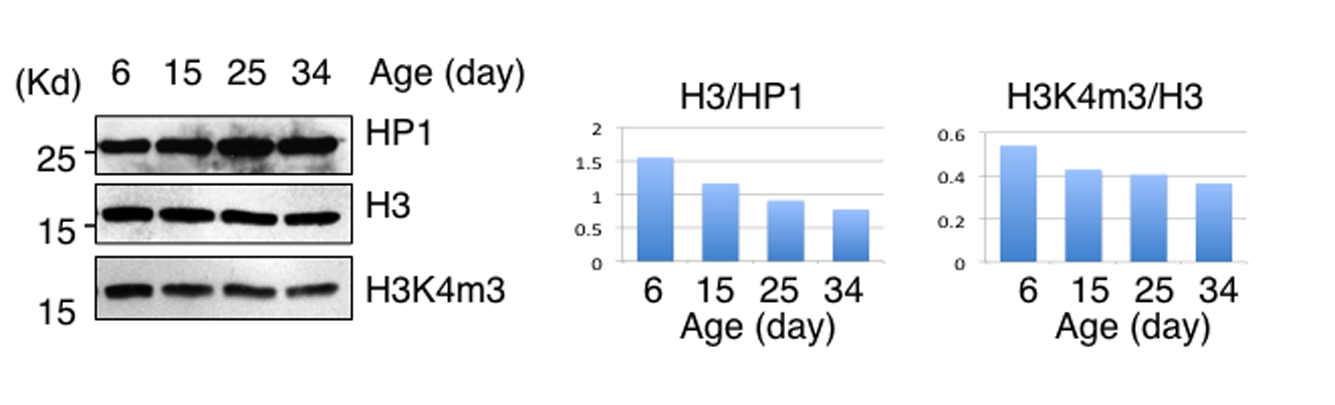

Supplement: Figure S4 — Levels of H3K4m3 at different ages. Male flies of indicated age (in days) were homogenized and the protein extracts were subjected to SDS-PAGE and blotted sequentially with antibodies for H3K4m3, HP1, and total H3. Representative images for one of the three experiments are shown. Intensity ratios are shown. Note that relative to HP1, total H3 levels decrease with age, and that H3K4m3 signals (relative to H3) also show moderate decrease with age. (TIF) [file pgen.1002473.s004.tif]
